# Supplementary figures and images for: Solute exchange through gap junctions lessens the adverse effects of inactivating mutations in metabolite-handling genes
Source: eLife. 2022 Sep 15;11:e78425. doi: 10.7554/eLife.78425 (PMC9534548; doi:10.7554/eLife.78425)

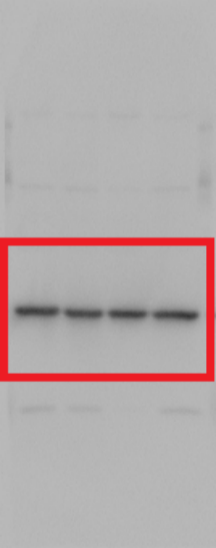

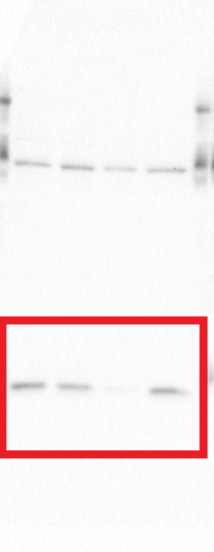

11111111

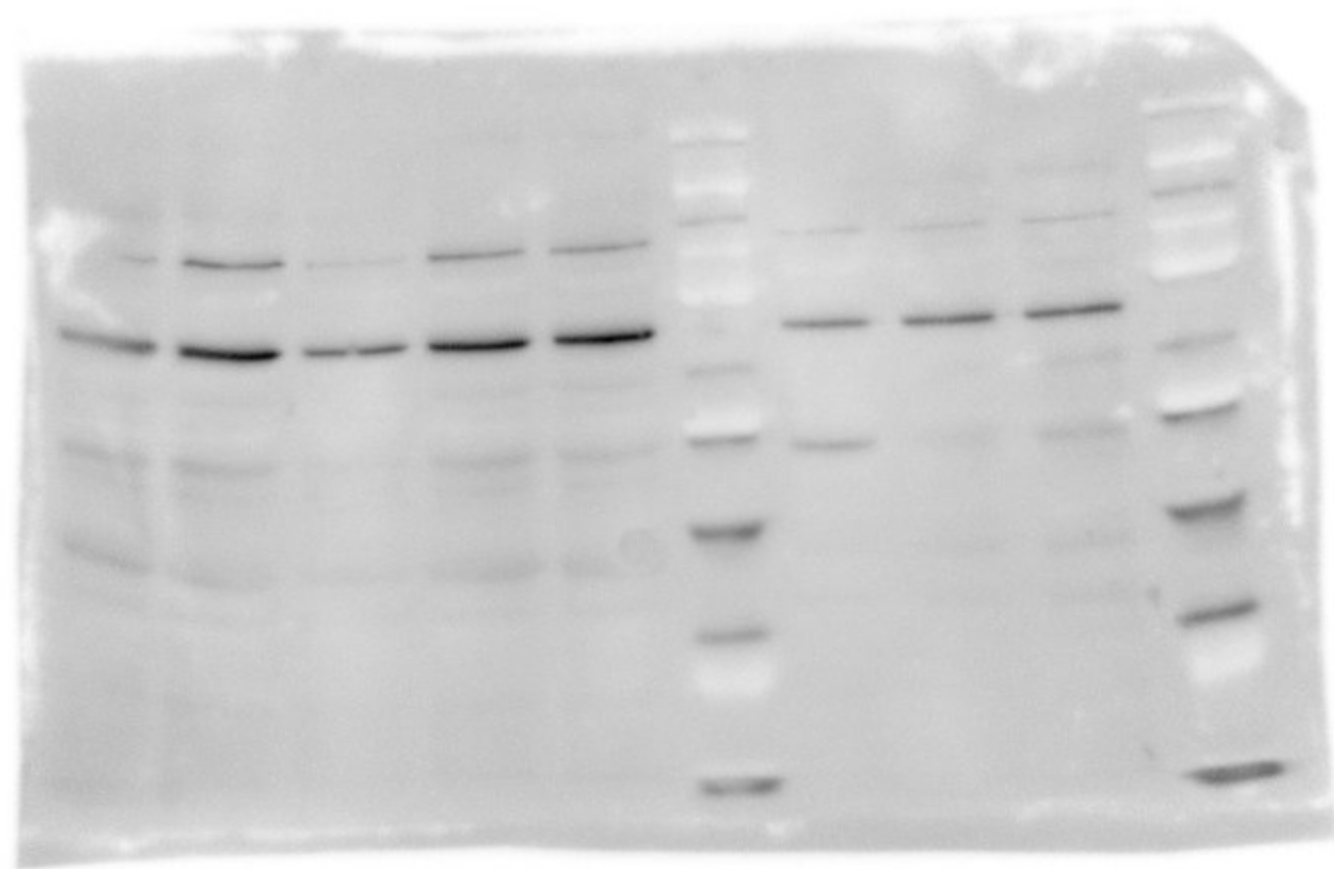

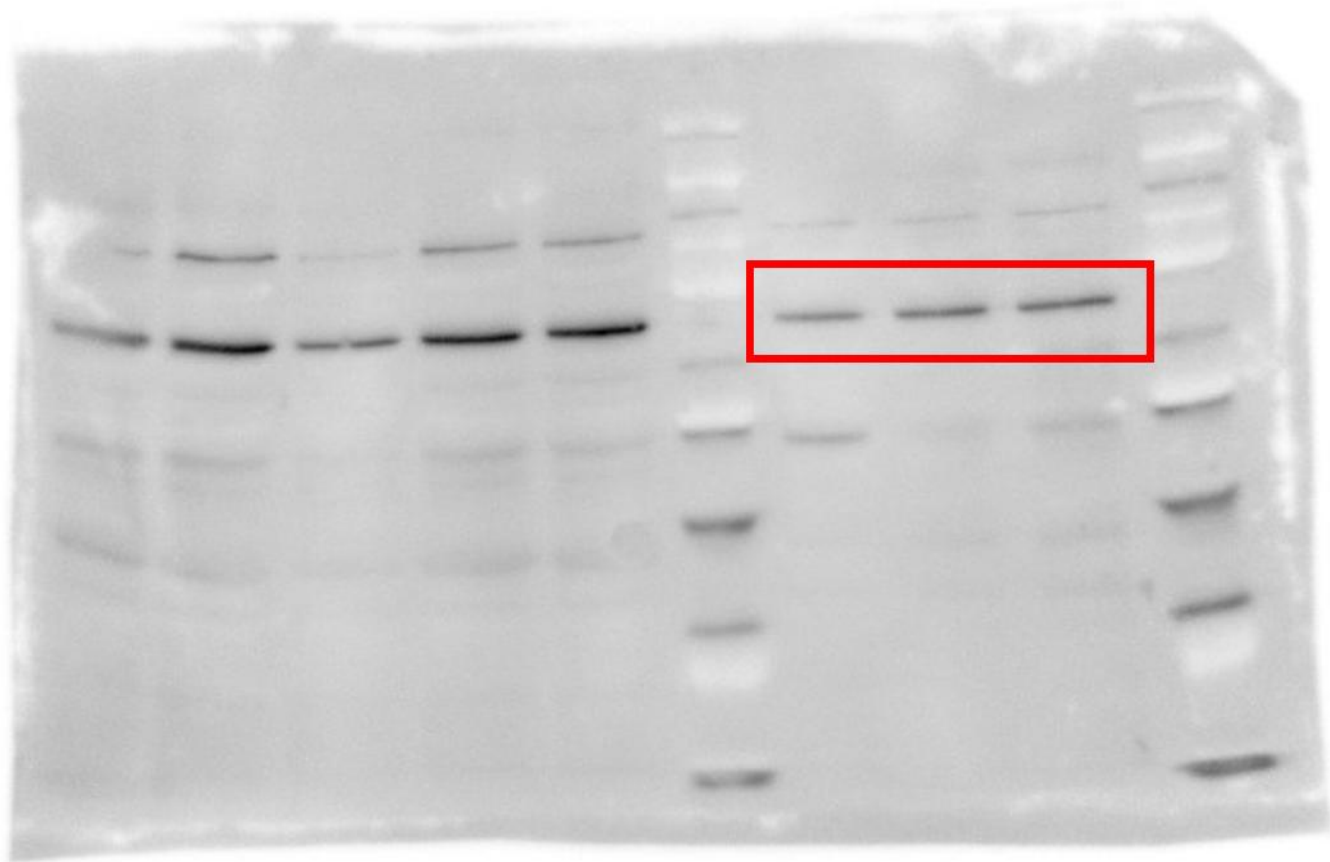

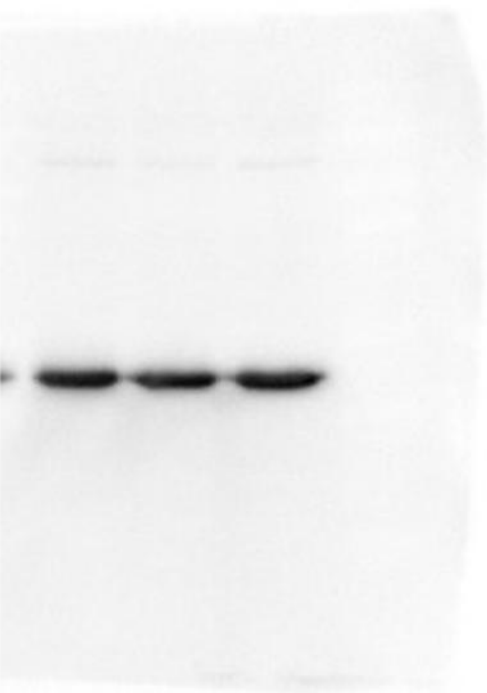

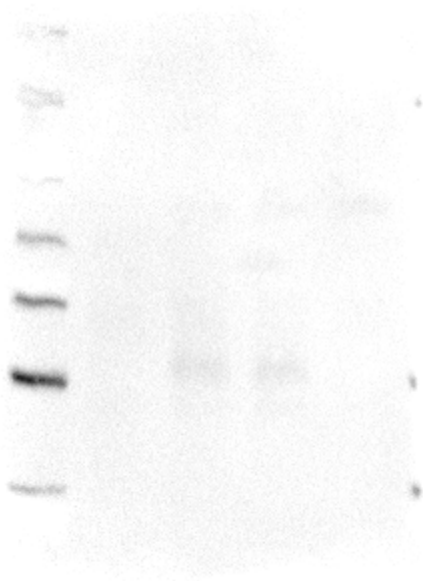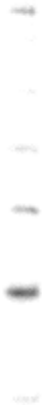

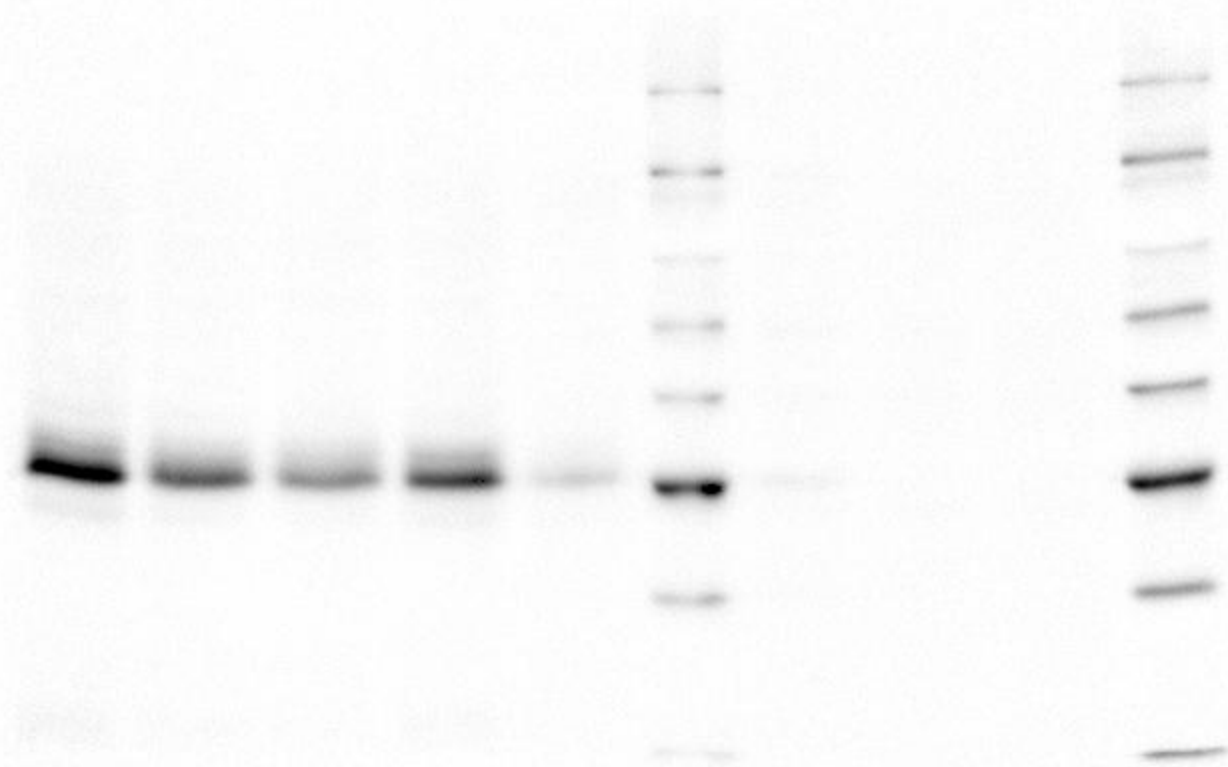

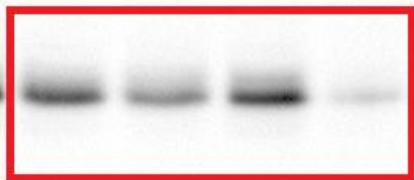

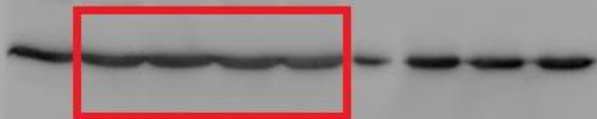

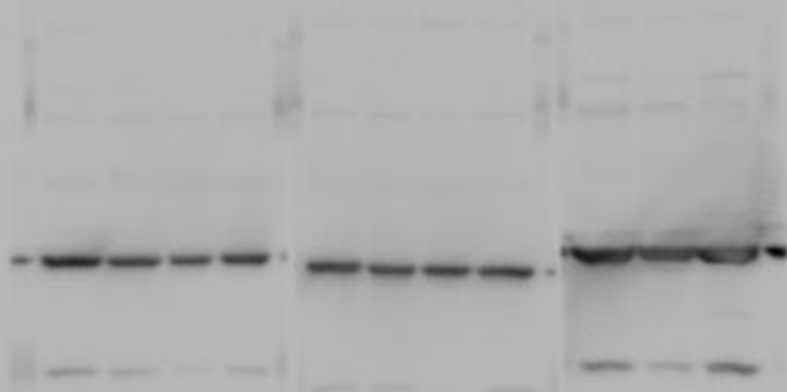

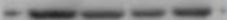

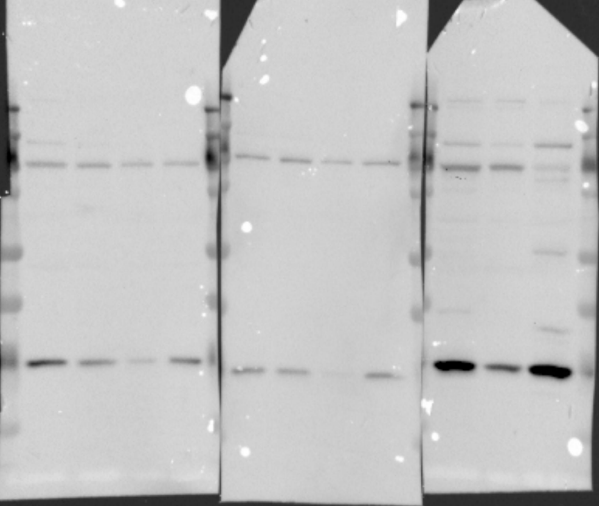

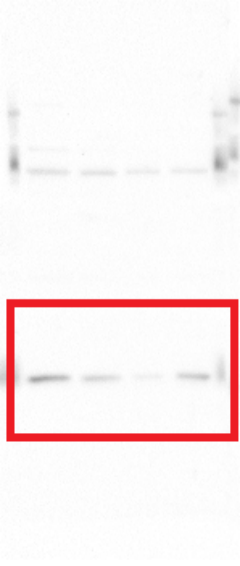

Supplement: Figure 2—figure supplement 1—source data 1. [file elife-78425-fig2-figsupp1-data1.zip › Full length blots.pdf]

Cx 43

75 -

50 -

37 -

25 -

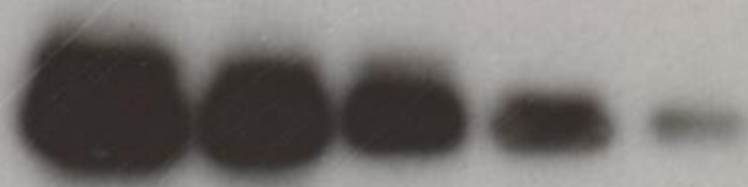

Cx43

75 -

50 -

37 -

25 -

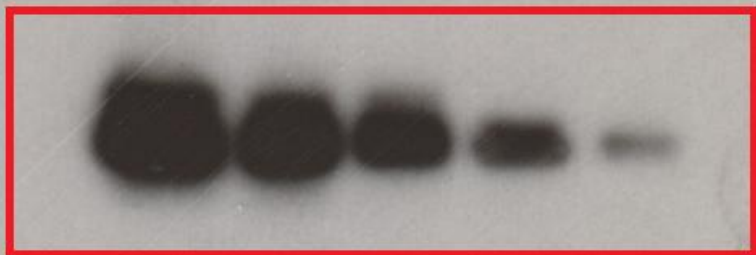

Supplement: Figure 2—figure supplement 2—source data 1. [file elife-78425-fig2-figsupp2-data1.zip › Figure 2-figure supp 2-source data 1.pdf]

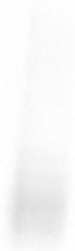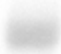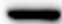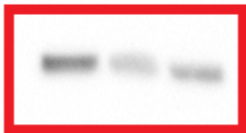

•

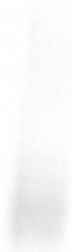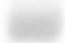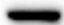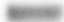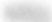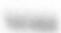

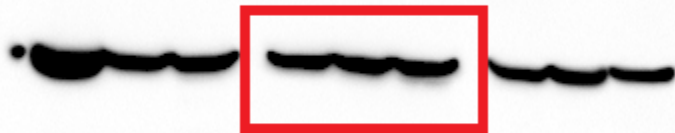

Supplement: Figure 6—figure supplement 3—source data 1. [file elife-78425-fig6-figsupp3-data1.zip › Figure 6-figure supp 3-source data 1.pdf]

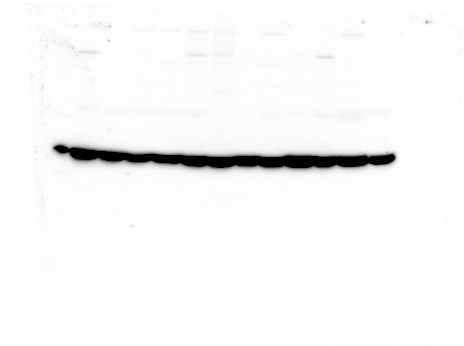

Supplement: Source data 1. [file elife-78425-data1.zip › revised/Figure 1-source data 1/Figure 1F-source data Actin.tif]

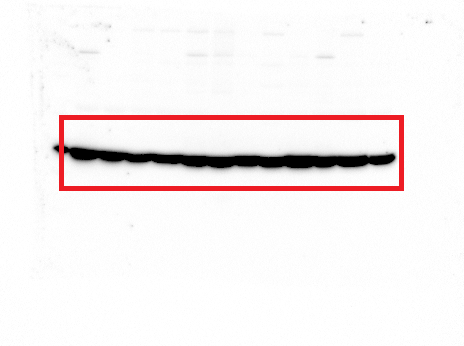

Supplement: Source data 1. [file elife-78425-data1.zip › revised/Figure 1-source data 1/Figure 1F-source data Actin_HIGHLIGHTED.tif]

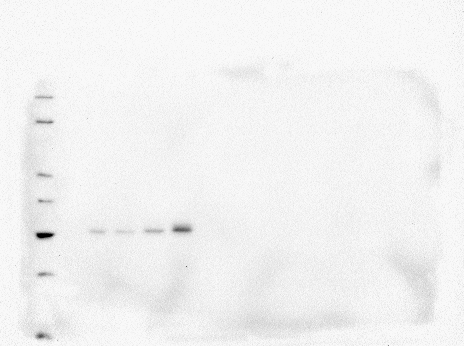

Supplement: Source data 1. [file elife-78425-data1.zip › revised/Figure 1-source data 1/Figure 1F-source data Cx43.tif]

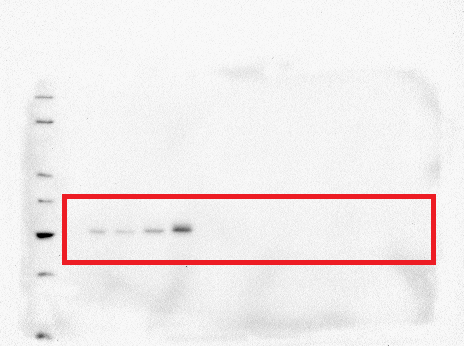

Supplement: Source data 1. [file elife-78425-data1.zip › revised/Figure 1-source data 1/Figure 1F-source data Cx43_HIGHLIGHTED.tif]

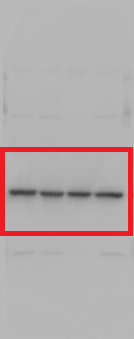

Supplement: Source data 1. [file elife-78425-data1.zip › revised/Figure 2-figure supplement 1-source data 1/Figure A_Actin_blot_for_Cx31_HIGHLIGHTED.tif]

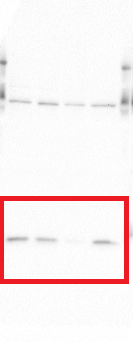

Supplement: Source data 1. [file elife-78425-data1.zip › revised/Figure 2-figure supplement 1-source data 1/Figure A_Cx31 blot_HIGHLIGHTED.tif]

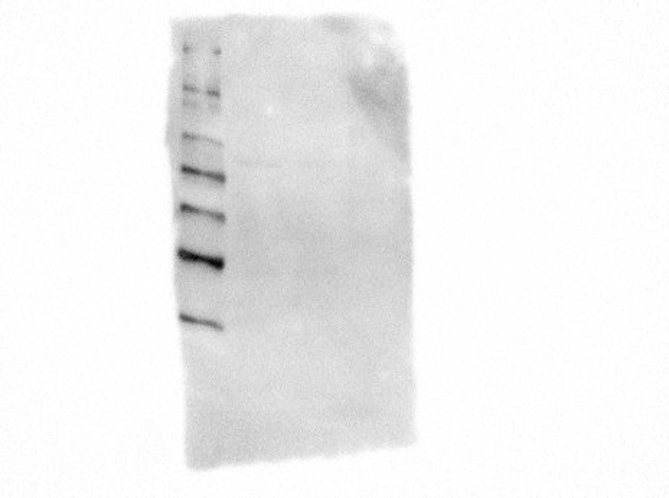

Supplement: Source data 1. [file elife-78425-data1.zip › revised/Figure 2-figure supplement 1-source data 1/Figure B- source data Cx43 DLD.tif]

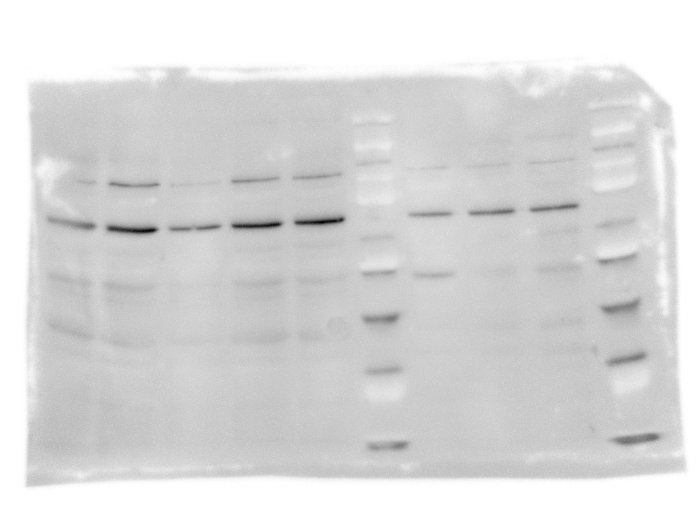

Supplement: Source data 1. [file elife-78425-data1.zip › revised/Figure 2-figure supplement 1-source data 1/Figure C - source data Cx26 DLD.tif]

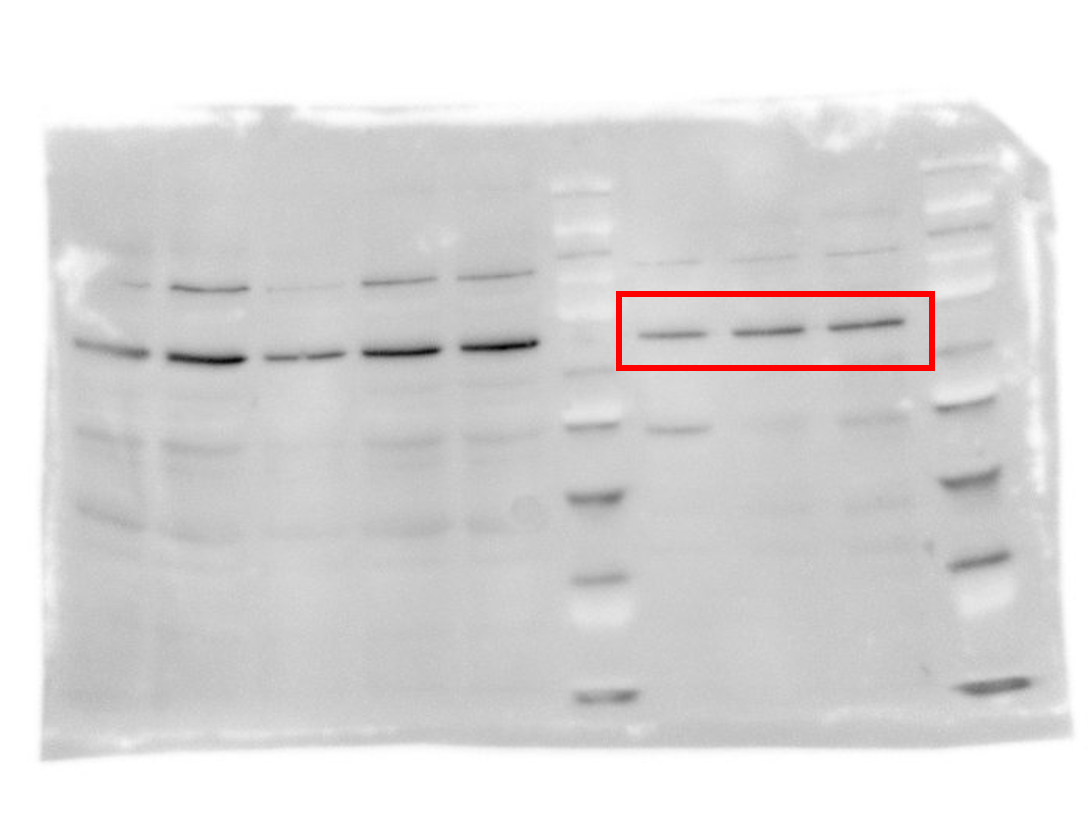

Supplement: Source data 1. [file elife-78425-data1.zip › revised/Figure 2-figure supplement 1-source data 1/Figure C-source data Cx26 DLD_HIGHLIGHTED.tif]

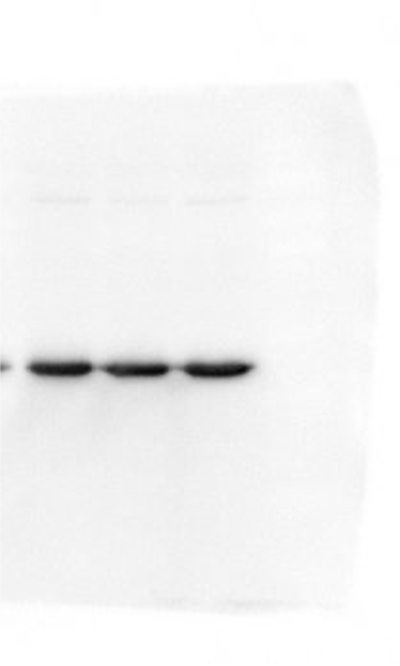

Supplement: Source data 1. [file elife-78425-data1.zip › revised/Figure 2-figure supplement 1-source data 1/Figure D - source data actin.tif]

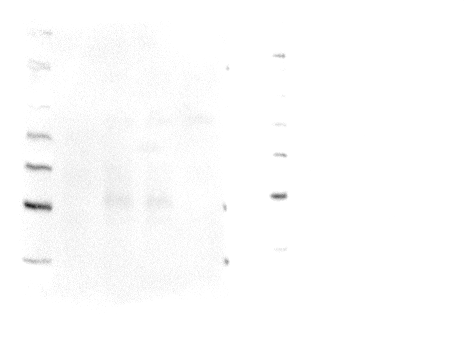

Supplement: Source data 1. [file elife-78425-data1.zip › revised/Figure 2-figure supplement 1-source data 1/Figure D - source data cx43 DLD.tif]

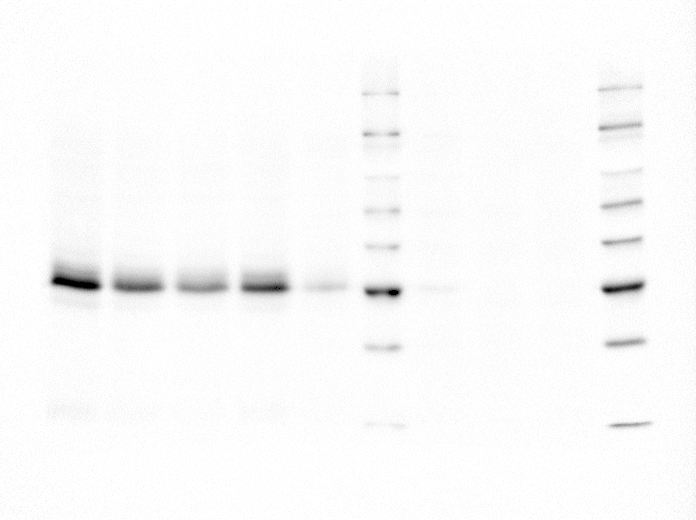

Supplement: Source data 1. [file elife-78425-data1.zip › revised/Figure 2-figure supplement 1-source data 1/Figure E - source data Cx43.tif]

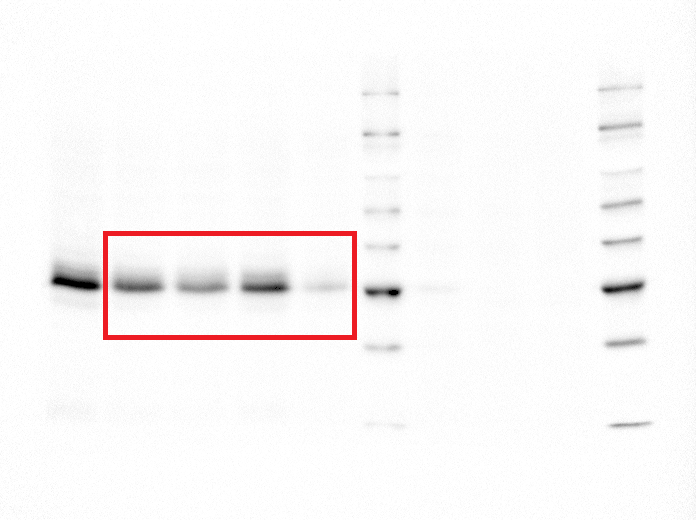

Supplement: Source data 1. [file elife-78425-data1.zip › revised/Figure 2-figure supplement 1-source data 1/Figure E - source data_Cx43_HIGHLIGHTED.tif]

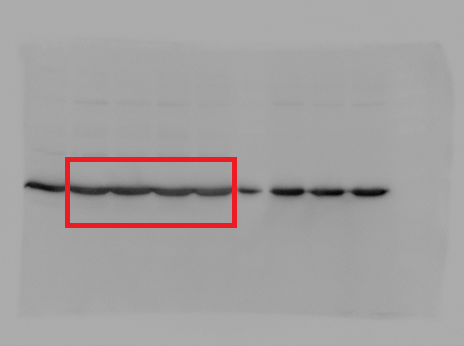

Supplement: Source data 1. [file elife-78425-data1.zip › revised/Figure 2-figure supplement 1-source data 1/Figure E_actin for Cx43_HIGHLIGHTED.tif]

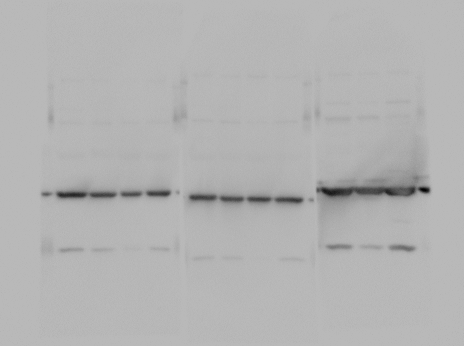

Supplement: Source data 1. [file elife-78425-data1.zip › revised/Figure 2-figure supplement 1-source data 1/Figure F - source data actin Cx31.tif]

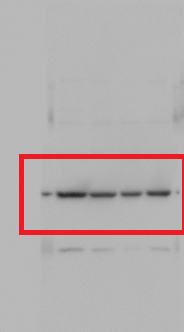

Supplement: Source data 1. [file elife-78425-data1.zip › revised/Figure 2-figure supplement 1-source data 1/Figure F - source data actin Cx31_HIGHLIGHTED.tif]

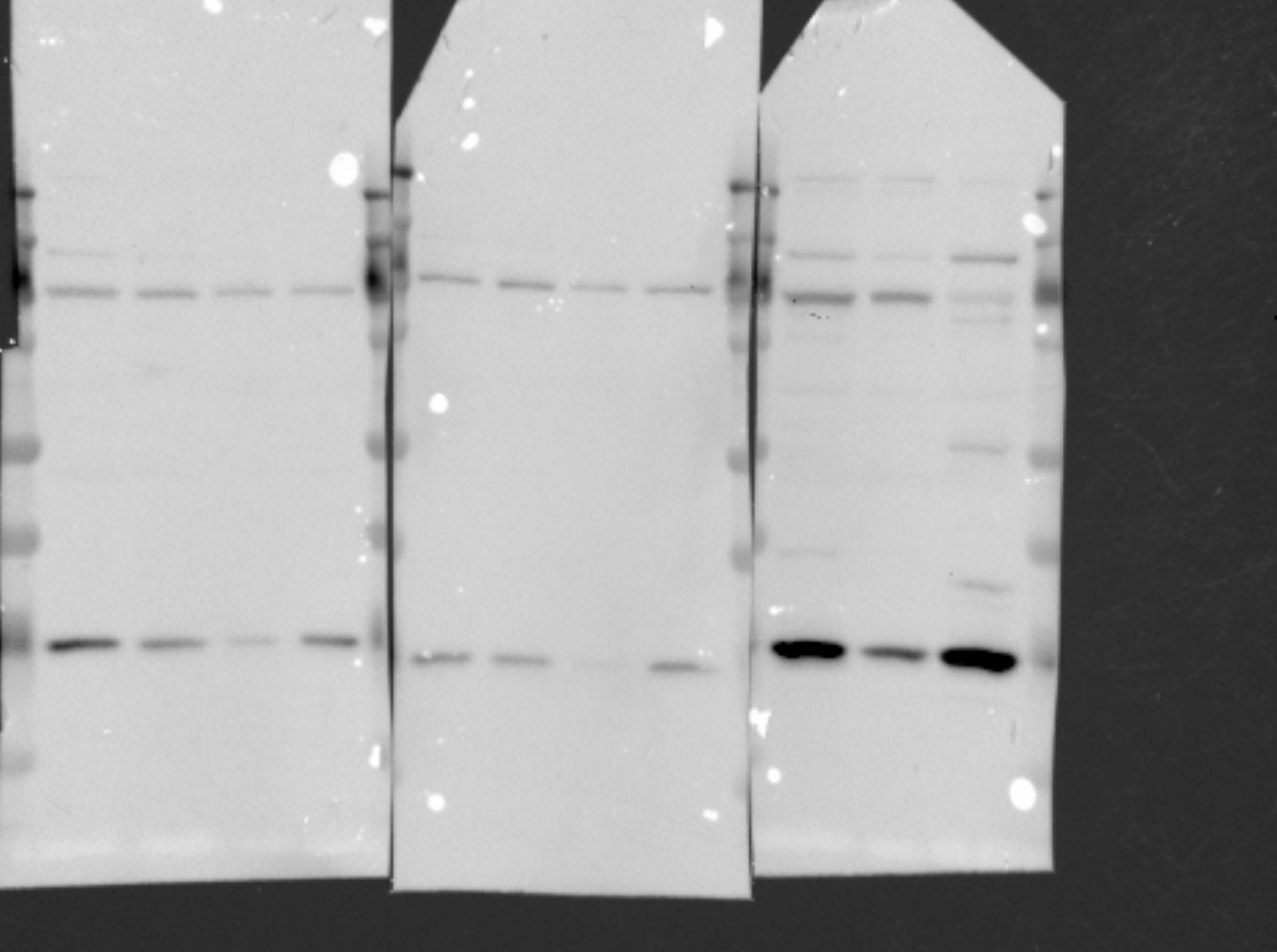

Supplement: Source data 1. [file elife-78425-data1.zip › revised/Figure 2-figure supplement 1-source data 1/Figure F - source data Cx31.tif]

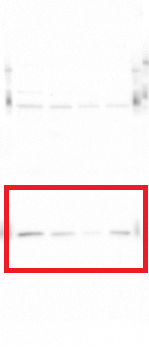

Supplement: Source data 1. [file elife-78425-data1.zip › revised/Figure 2-figure supplement 1-source data 1/Figure F_Cx31 blot_HIGHLIGHTED.tif]

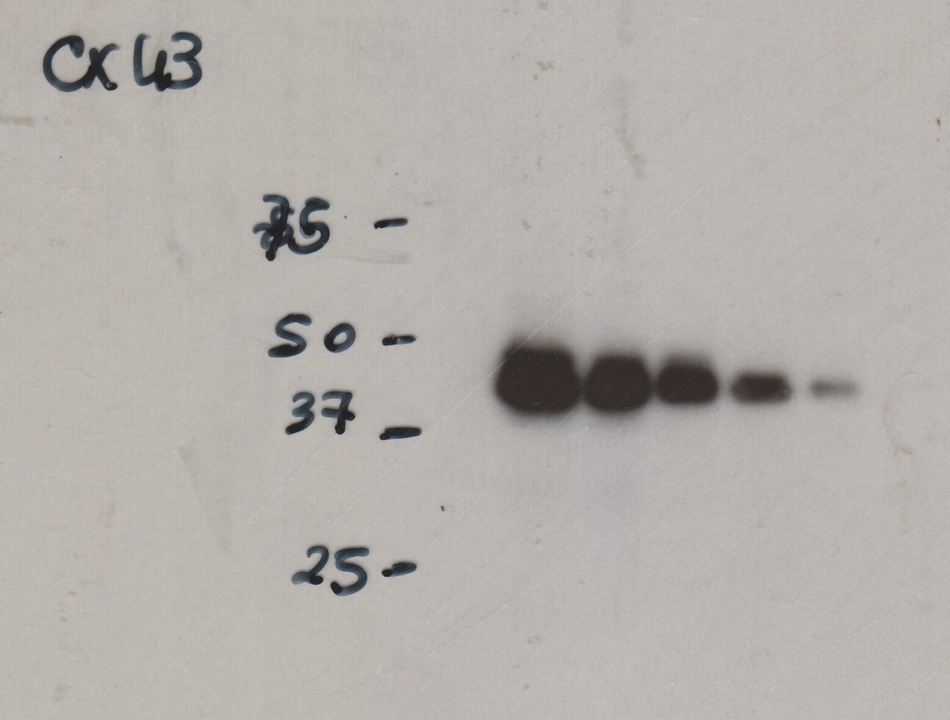

Supplement: Source data 1. [file elife-78425-data1.zip › revised/Figure 2-figure supplement 2-source data 1/Figure source data Cx43siRNA.tif]

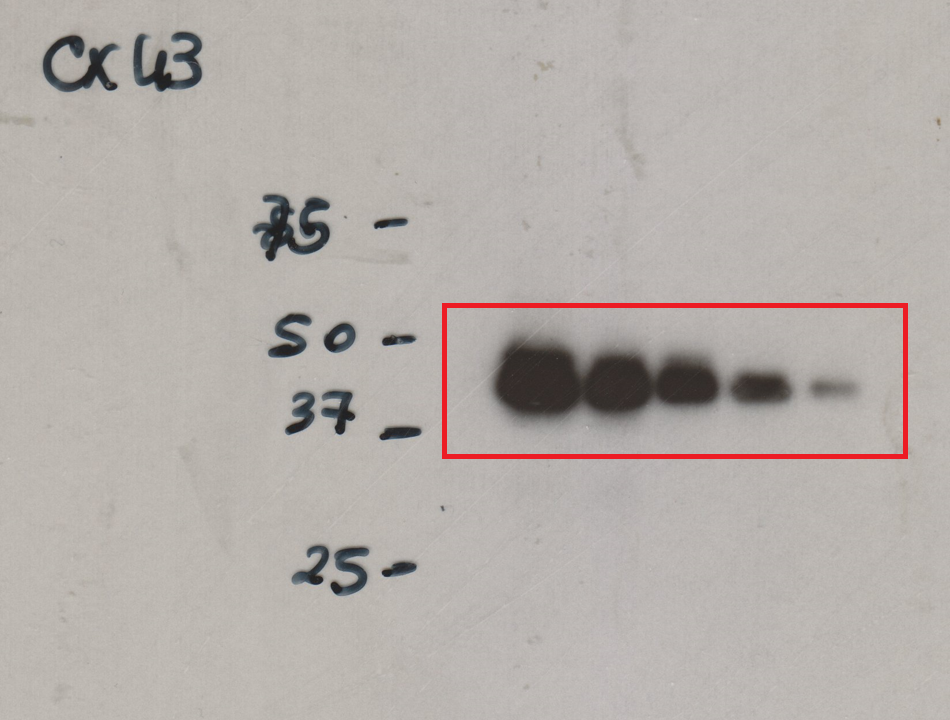

Supplement: Source data 1. [file elife-78425-data1.zip › revised/Figure 2-figure supplement 2-source data 1/Figure source data Cx43siRNA_HIGHLIGHTED.tif]

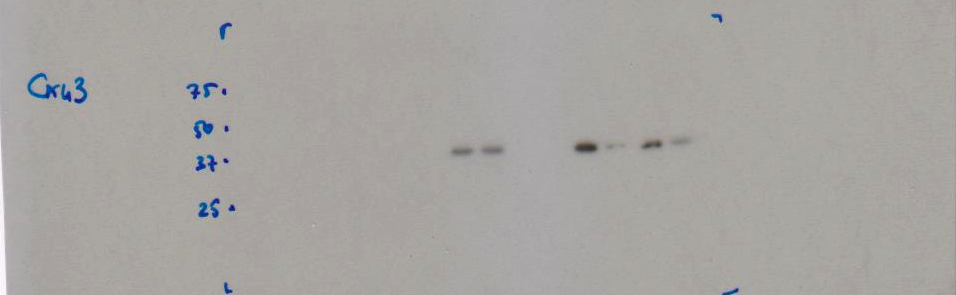

Supplement: Source data 1. [file elife-78425-data1.zip › revised/Figure 2-source data 1/Figure 2H - source data Cx43.tif]

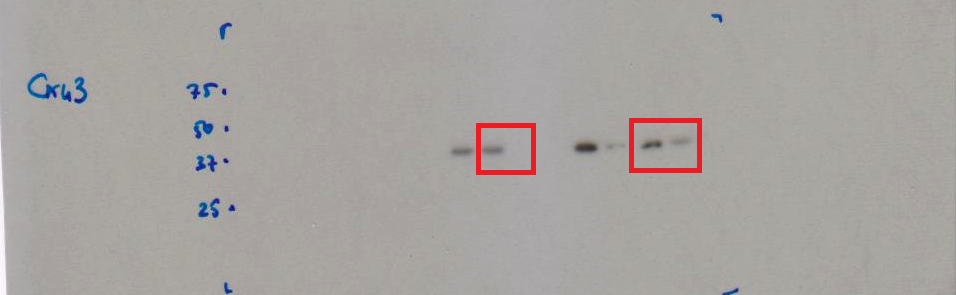

Supplement: Source data 1. [file elife-78425-data1.zip › revised/Figure 2-source data 1/Figure 2H - source data Cx43_HIGHLIGHTED.tif]

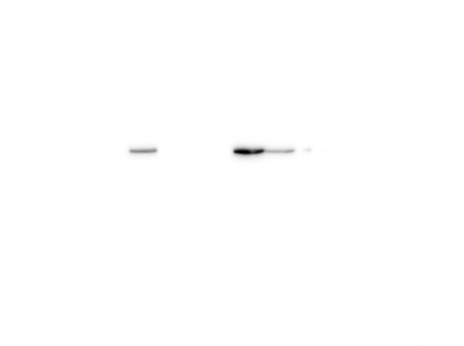

Supplement: Source data 1. [file elife-78425-data1.zip › revised/Figure 2-source data 1/Figure 2I - source data Cx26KO SW1222 .tif]

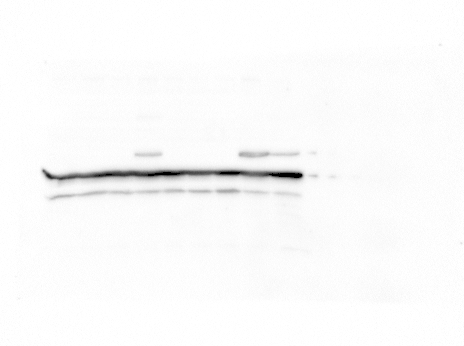

Supplement: Source data 1. [file elife-78425-data1.zip › revised/Figure 2-source data 1/Figure 2I- source data GAPDH SW1222.tif]

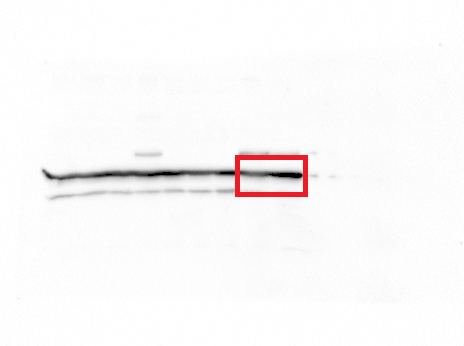

Supplement: Source data 1. [file elife-78425-data1.zip › revised/Figure 2-source data 1/Figure 2I- source data GAPDH SW1222_HIGHLIGHTED.tif]

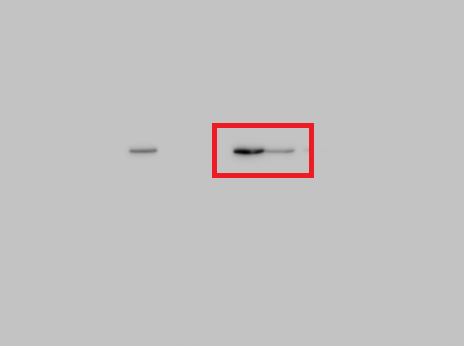

Supplement: Source data 1. [file elife-78425-data1.zip › revised/Figure 2-source data 1/Figure 2I-source data Cx26KO SW1222_HIGHLIGHTED.tif]

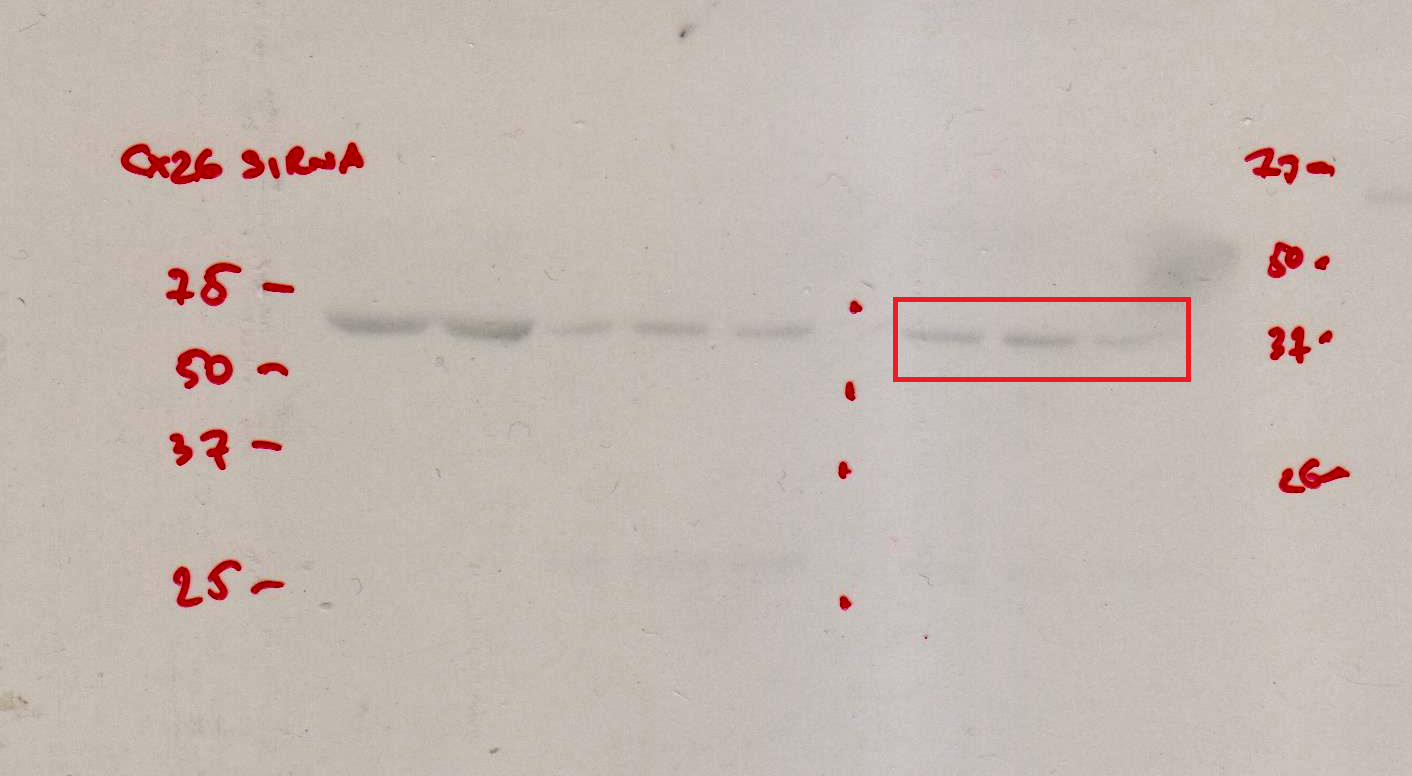

Supplement: Source data 1. [file elife-78425-data1.zip › revised/Figure 2-source data 1/Figure 2I-source data Cx26siRNA DLD_HIGHLIGHTED.tif]

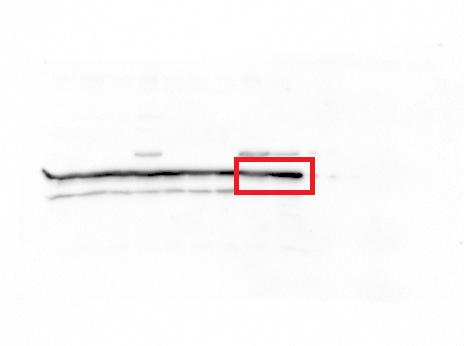

Supplement: Source data 1. [file elife-78425-data1.zip › revised/Figure 2-source data 1/Figure 2I-source data GAPDH SW1222_HIGHLIGHTED.tif]

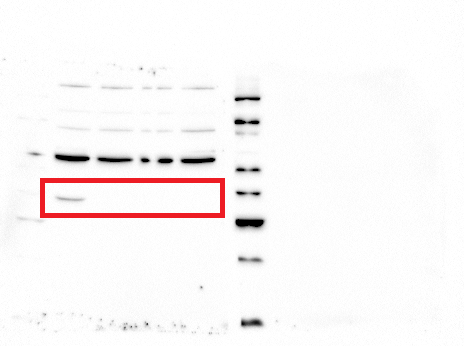

Supplement: Source data 1. [file elife-78425-data1.zip › revised/Figure 2-source data 1/Figure 2K-source data Cx26 KO DLD_HIGHLIGHTED.tif]

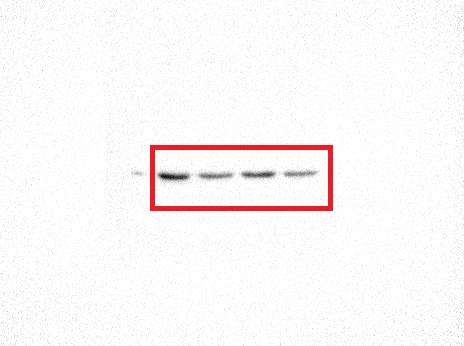

Supplement: Source data 1. [file elife-78425-data1.zip › revised/Figure 2-source data 1/Figure 2K-source data GAPDH Cx26KO_HIGHLIGHTED.tif]

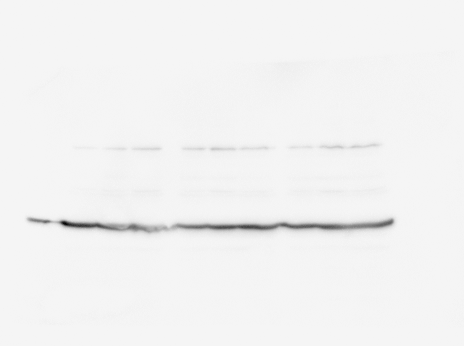

Supplement: Source data 1. [file elife-78425-data1.zip › revised/Figure 5-source data 1/Figure 5 - source data actin.tif]

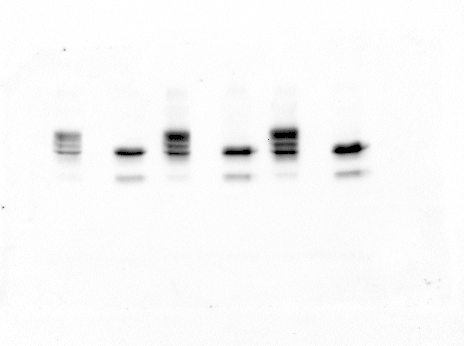

Supplement: Source data 1. [file elife-78425-data1.zip › revised/Figure 5-source data 1/Figure 5 - source data NHE1 .tif]

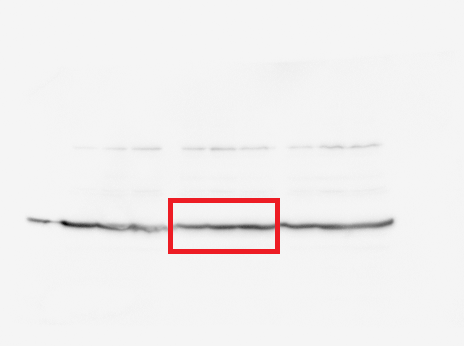

Supplement: Source data 1. [file elife-78425-data1.zip › revised/Figure 5-source data 1/Figure 5-source data actin_HIGHLIGHTED.tif]

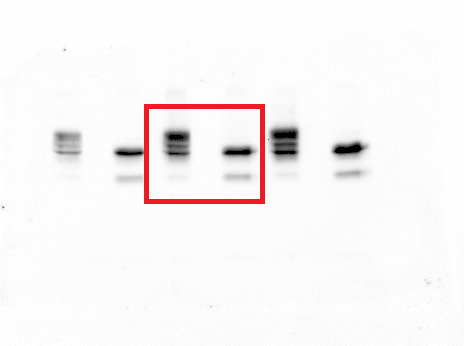

Supplement: Source data 1. [file elife-78425-data1.zip › revised/Figure 5-source data 1/Figure 5-source data NHE1_HIGHLIGHTED.tif]

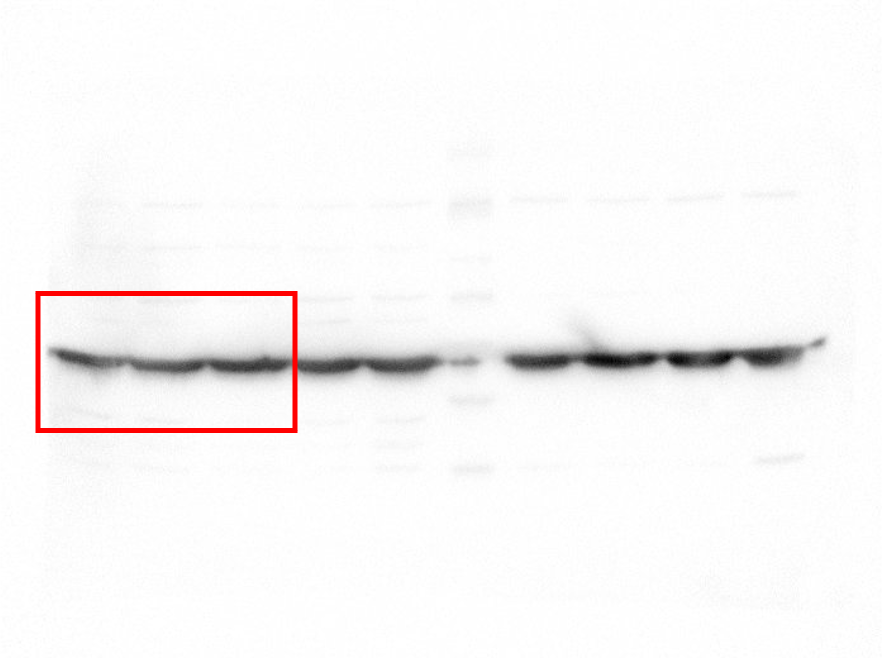

Supplement: Source data 1. [file elife-78425-data1.zip › revised/Figure 6-figure supplement 3-source data 1/Figure A - source data actin C10_HIGHLIGHTED.tif]

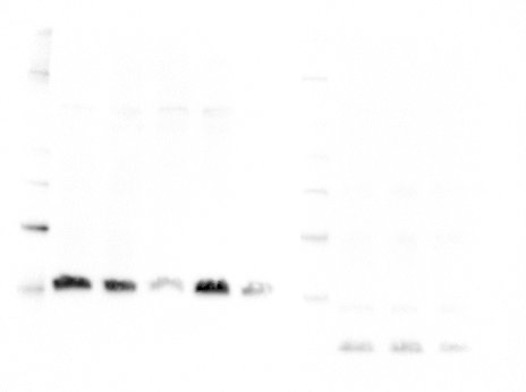

Supplement: Source data 1. [file elife-78425-data1.zip › revised/Figure 6-figure supplement 3-source data 1/Figure A - source data ALDOA C10.tif]

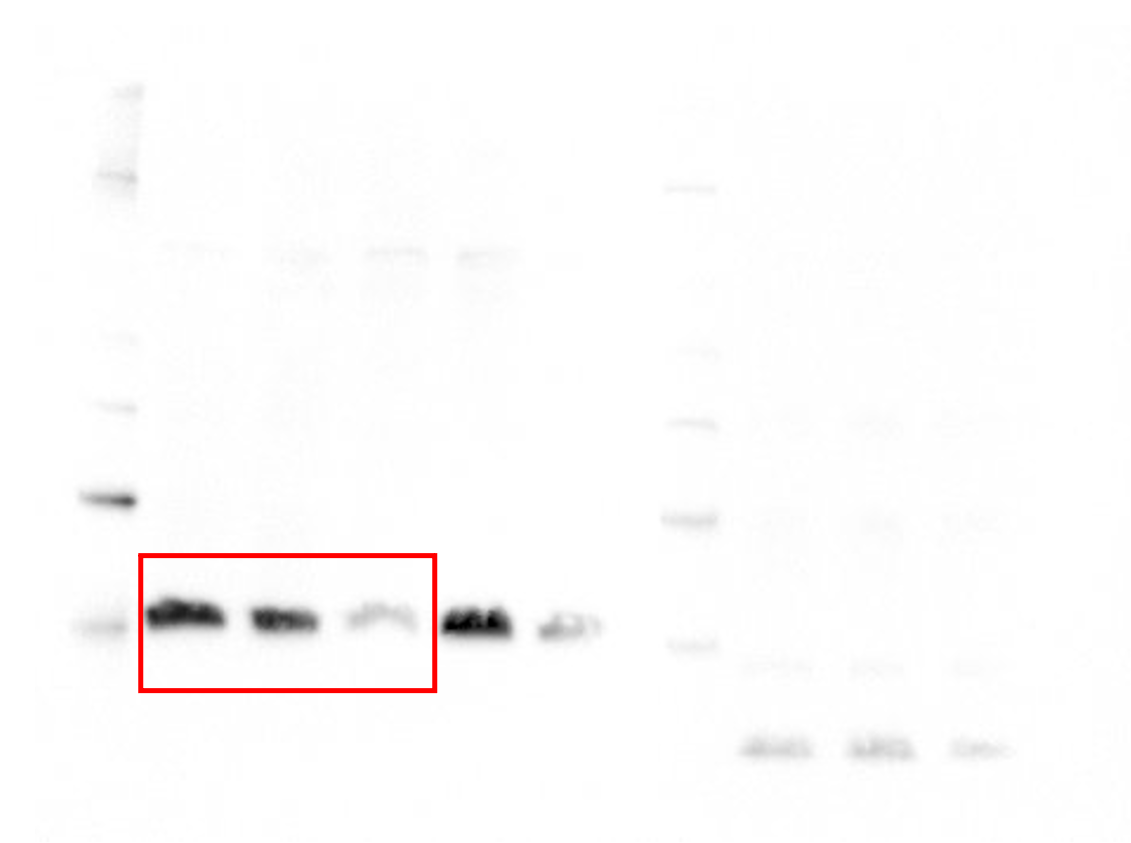

Supplement: Source data 1. [file elife-78425-data1.zip › revised/Figure 6-figure supplement 3-source data 1/Figure A - source data ALDOA C10_HIGHLIGHTED.tif]

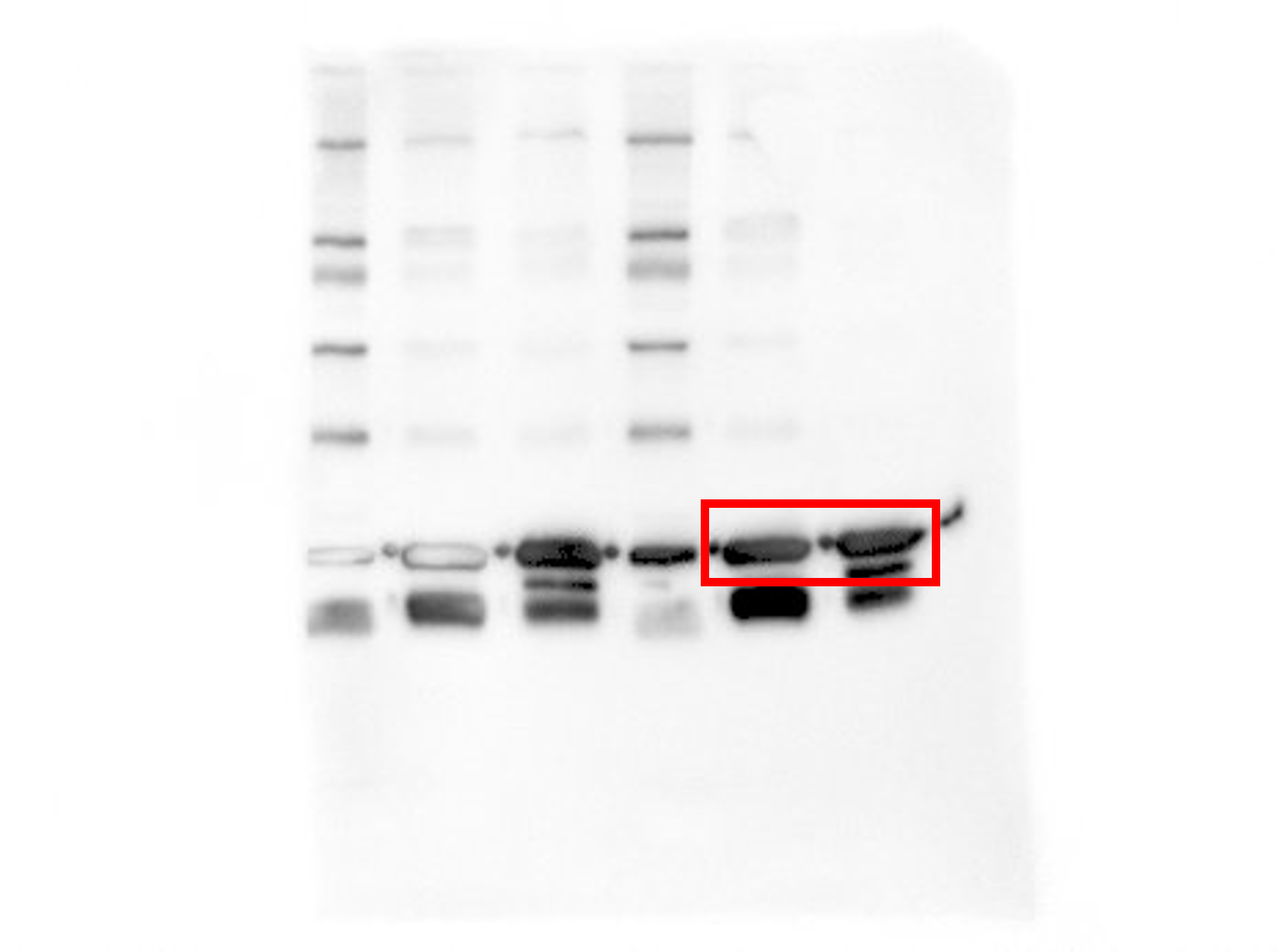

Supplement: Source data 1. [file elife-78425-data1.zip › revised/Figure 6-figure supplement 3-source data 1/Figure B-source data actin NCIH747.tif]

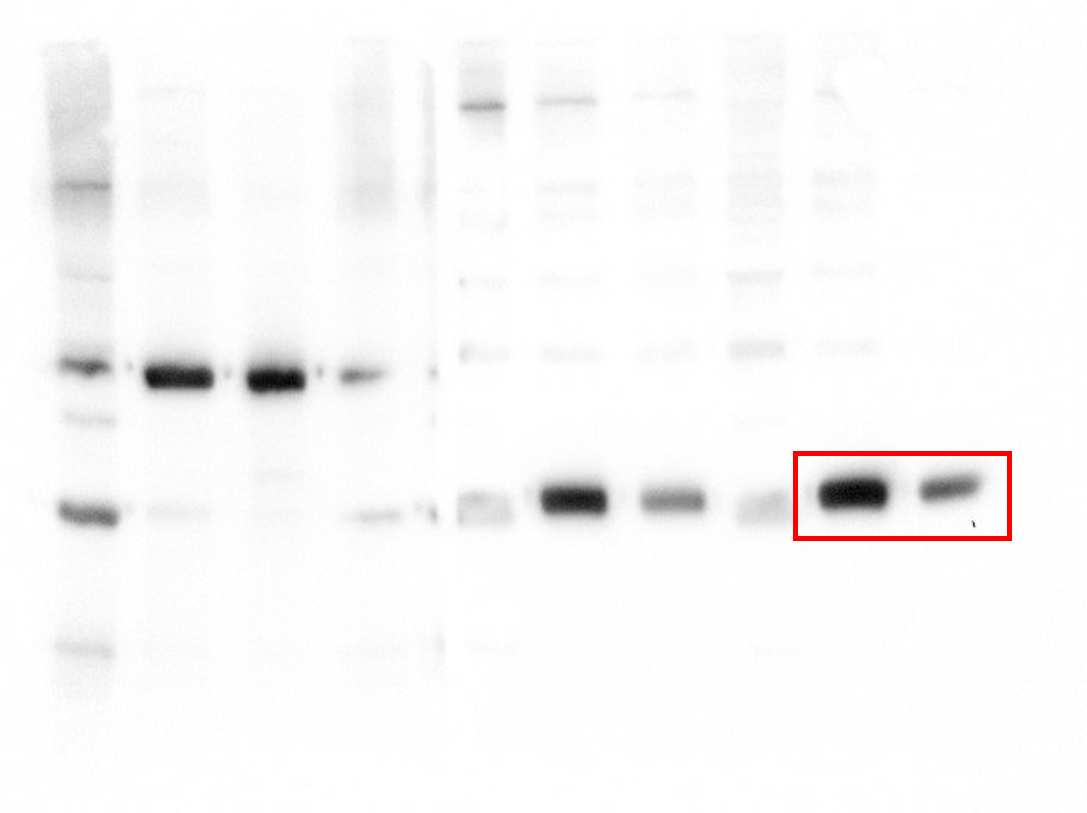

Supplement: Source data 1. [file elife-78425-data1.zip › revised/Figure 6-figure supplement 3-source data 1/Figure B-source data ALDOA NCIH747.tif]

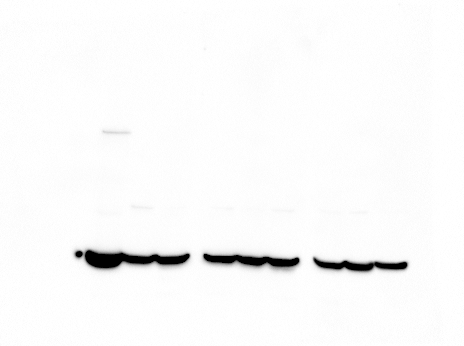

Supplement: Source data 1. [file elife-78425-data1.zip › revised/Figure 6-source data 1/Figure 6 - source data actin.tif]

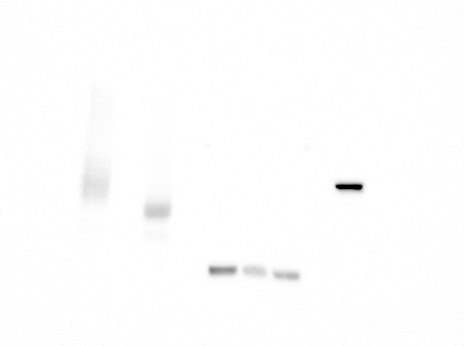

Supplement: Source data 1. [file elife-78425-data1.zip › revised/Figure 6-source data 1/Figure 6 - source data aldoa.tif]

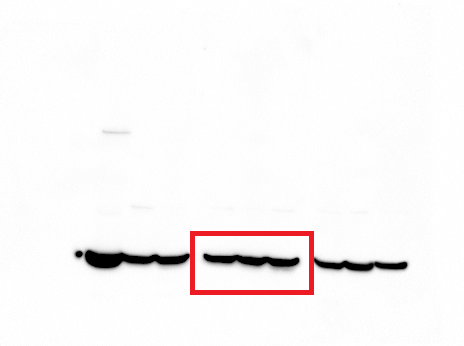

Supplement: Source data 1. [file elife-78425-data1.zip › revised/Figure 6-source data 1/Figure 6-source data actin_HIGHLIGHTED.tif]

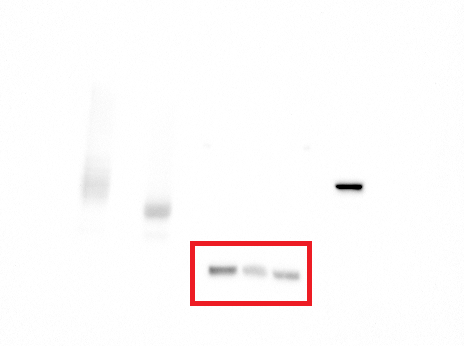

Supplement: Source data 1. [file elife-78425-data1.zip › revised/Figure 6-source data 1/Figure 6-source data aldoa_HIGHLIGHTED.tif]

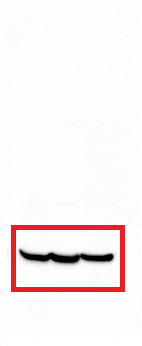

Supplement: Source data 1. [file elife-78425-data1.zip › revised/Figure 7-source data 1/Figure 7-source data actin_HIGHLIGHTED.tif]

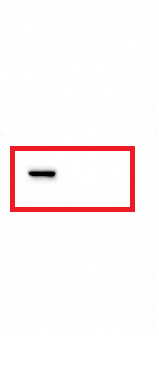

Supplement: Source data 1. [file elife-78425-data1.zip › revised/Figure 7-source data 1/Figure 7-source data NDUFS1_HIGHLIGHTED.tif]

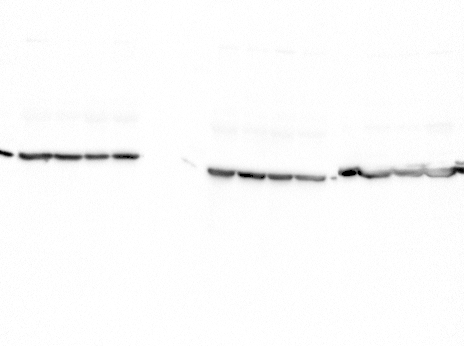

Supplement: Source data 1. [file elife-78425-data1.zip › revised/Figure 8-source data 1/Figure 8A_Actin_blot_for_Cx26.tif]

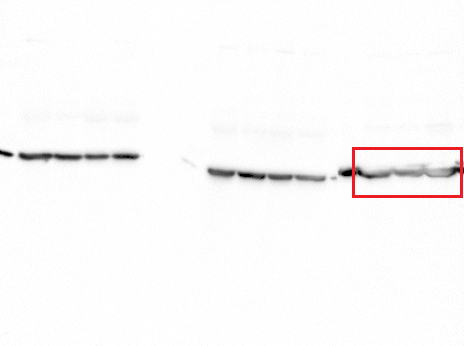

Supplement: Source data 1. [file elife-78425-data1.zip › revised/Figure 8-source data 1/Figure 8A_Actin_blot_for_Cx26_HIGHLIGHTED.tif]

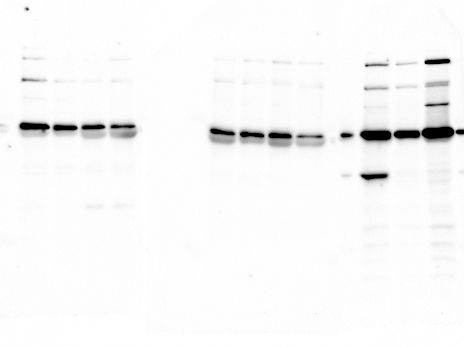

Supplement: Source data 1. [file elife-78425-data1.zip › revised/Figure 8-source data 1/Figure 8B_Cx26_blot_for_Cx26.tif]

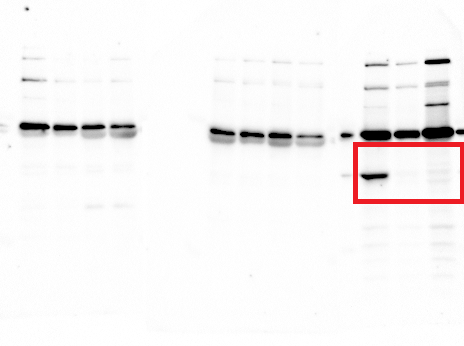

Supplement: Source data 1. [file elife-78425-data1.zip › revised/Figure 8-source data 1/Figure 8B_Cx26_blot_for_Cx26_HIGHLIGHTED.tif]
