# Supplementary material for: Solute exchange through gap junctions lessens the adverse effects of inactivating mutations in metabolite-handling genes
Source: eLife. 2022 Sep 15;11:e78425. doi: 10.7554/eLife.78425 (PMC9534548; doi:10.7554/eLife.78425)
Supplement: Supplementary file 1. [file elife-78425-supp1.docx]

| Line | **FRAP perme-ability** | Log_2_ expression | | | | | | |
| --- | --- | --- | --- | --- | --- | --- | --- | --- |
|  |  | *GJA1* | *GJA3* | *GJB1* | *GJB2* | *GJB3* | *GJB5* | *GJC1* |
| LS174T | **0.0492** | 8.4809 | 3.60227 | 7.01475 | 9.44016 | 7.4432 | 5.48909 | 4.12497 |
| OXCO1 | **0.0076** | 10.118 | 8.1849 | 6.32904 | 10.1954 | 9.30414 | 5.41039 | 8.80613 |
| CC20 | **-0.0139** | 10.3215 | 5.06252 | 6.61397 | 8.0947 | 6.72236 | 5.00941 | 11.1327 |
| CACO2 | **0.3372** | 10.699 | 3.95102 | 6.4605 | 6.47709 | 6.26461 | 5.22355 | 9.62387 |
| LOVO | **0.2081** | 10.6907 | 6.14784 | 6.37122 | 9.98964 | 10.0456 | 5.7526 | 7.8109 |
| C10 | **0.0893** | 11.49956 | 3.72555 | 6.882 | 4.8782 | 8.87293 | 6.67993 | 8.23218 |
| SW1222 | **0.4343** | 4.87689 | 3.52786 | 8.30044 | 7.74669 | 7.44094 | 5.3939 | 5.0809 |
| HT29 | **0.1686** | 4.69231 | 4.06969 | 8.25277 | 9.35743 | 9.14403 | 4.99819 | 3.68293 |
| DLD1 | **0.8675** | 5.00976 | 5.62883 | 6.47561 | 10.3706 | 9.38289 | 5.30895 | 4.16066 |
| SNU1235 | **0.7937** | 3.84928 | 6.51597 | 6.55618 | 12.024 | 7.44414 | 4.51103 | 3.6195 |
| SW948 | **0.7497** | 3.57366 | 4.99115 | 6.82146 | 11.0571 | 8.43129 | 5.36555 | 7.68649 |
| HDC9 | **0.1572** | 6.34948 | 4.68167 | 6.35626 | 6.59396 | 6.90614 | 4.06943 | 8.16224 |
| NCIH747 | **0.0016** | 3.80565 | 3.78693 | 6.51244 | 5.46257 | 8.98933 | 4.93663 | 9.73081 |
| HCT116 | **0.0607** | 5.1623 | 5.94231 | 6.45528 | 9.39629 | 8.15557 | 5.52061 | 9.96134 |
| Pearson's correlation (**FRAP** v expression) | | -0.501 | +0.131 | +0.0390 | +0.537 | +0.0167 | -0.167 | -0.542 |
