# Supplementary material for: Solute exchange through gap junctions lessens the adverse effects of inactivating mutations in metabolite-handling genes
Source: eLife. 2022 Sep 15;11:e78425. doi: 10.7554/eLife.78425 (PMC9534548; doi:10.7554/eLife.78425)
Supplement: Transparent reporting form [file elife-78425-transrepform1.docx]

***eLife’s* transparent reporting form**

We encourage authors to provide detailed information *within their submission* to facilitate the interpretation and replication of experiments. Authors can upload supporting documentation to indicate the use of appropriate reporting guidelines for health-related research (see [EQUATOR Network](http://www.equator-network.org/%20)), life science research (see the [BioSharing Information Resource](https://biosharing.org/)), or the [ARRIVE guidelines](http://www.plosbiology.org/article/info:doi/10.1371/journal.pbio.1000412) for reporting work involving animal research. Where applicable, authors should refer to any relevant reporting standards documents in this form.

If you have any questions, please consult our Journal Policies and/or contact us: [editorial@elifesciences.org](mailto:editorial@elifesciences.org).

**Sample-size estimation**

- You should state whether an appropriate sample size was computed when the study was being designed
- You should state the statistical method of sample size computation and any required assumptions
- If no explicit power analysis was used, you should describe how you decided what sample (replicate) size (number) to use

Please outline where this information can be found within the submission (e.g., sections or figure legends), or explain why this information doesn’t apply to your submission:

Data presented are from cell lines, and figure legends outline the number of biological repeats (independent platings) and technical repeats (e.g. wells of the same series), and in some cases, further divided into individual cells (typically in the order of 20-100 per run). Sample size was at least 4 biological repeats (or more) with 3-4 technical repeats each. Work on cell lines minimized match effect. For in vivo experiments, xenografts with two conditions were paired (left, right flank) and 6 animals were used for study (none excluded).

**Replicates**

- You should report how often each experiment was performed
- You should include a definition of biological versus technical replication
- The data obtained should be provided and sufficient information should be provided to indicate the number of independent biological and/or technical replicates
- If you encountered any outliers, you should describe how these were handled
- Criteria for exclusion/inclusion of data should be clearly stated
- High-throughput sequence data should be uploaded before submission, with a private link for reviewers provided (these are available from both GEO and ArrayExpress)

Please outline where this information can be found within the submission (e.g., sections or figure legends), or explain why this information doesn’t apply to your submission:

Each legend reports the number of biological repeats, technical repeats, and (in the case of single-cell assays), the number of cells from which data were collected. Where indicated, hierarchical analyses were performed for data that showed evidence of clustering. No outliers were removed. Microarray data presented have been published previously and are re-analysed herein.

**Statistical reporting**

- Statistical analysis methods should be described and justified
- Raw data should be presented in figures whenever informative to do so (typically when N per group is less than 10)
- For each experiment, you should identify the statistical tests used, exact values of N, definitions of center, methods of multiple test correction, and dispersion and precision measures (e.g., mean, median, SD, SEM, confidence intervals; and, for the major substantive results, a measure of effect size (e.g., Pearson's r, Cohen's d)
- Report exact p-values wherever possible alongside the summary statistics and 95% confidence intervals. These should be reported for all key questions and not only when the p-value is less than 0.05.

Please outline where this information can be found within the submission (e.g., sections or figure legends), or explain why this information doesn’t apply to your submission:

For N<10, we present raw data together with summary statistics (mean+/-sem). For larger datasets, typically measured at cell-level, we present summary statistics. Legends indicate the tests used: 2x2 tables, Pearson’s correlation, two-way ANOVA and t-test (paired or unpaired, as indicated). Significance level was set to 0.05. Where technical repeats were obtained, hierarchical (nested) analyses were performed to avoid pseudoreplication error.

(For large datasets, or papers with a very large number of statistical tests, you may upload a single table file with tests, Ns, etc., with reference to sections in the manuscript.)

**Group allocation**

- Indicate how samples were allocated into experimental groups (in the case of clinical studies, please specify allocation to treatment method); if randomization was used, please also state if restricted randomization was applied
- Indicate if masking was used during group allocation, data collection and/or data analysis

Please outline where this information can be found within the submission (e.g., sections or figure legends), or explain why this information doesn’t apply to your submission:

Allocation did not apply to this cellular study. Randomisation was implemented in terms of order of measurements, where possible (e.g. imaging WT or KO). For animal work, WT DLD1 cells were injected into the left flank by convention. The opposite flank bore the KO cells.

**Additional data files (“source data”)**

- We encourage you to upload relevant additional data files, such as numerical data that are represented as a graph in a figure, or as a summary table
- Where provided, these should be in the most useful format, and they can be uploaded as “Source data” files linked to a main figure or table
- Include model definition files including the full list of parameters used
- Include code used for data analysis (e.g., R, MatLab)
- Avoid stating that data files are “available upon request”

Please indicate the figures or tables for which source data files have been provided:

We provide additional analyses in the supplement. Source data are included as excel sheets for numerical data presented in the main figures.
